# The Neuromuscular Disorder Mediated by Extracellular Vesicles in Amyotrophic Lateral Sclerosis

**DOI:** 10.3390/cimb46060358

**Published:** 2024-06-14

**Authors:** Elisabetta Carata, Marco Muci, Simona Di Giulio, Tiziano Di Giulio, Stefania Mariano, Elisa Panzarini

**Affiliations:** 1 Department of Biological and Environmental Sciences and Technologies, University of Salento, 73100 Lecce, Italy; elisabetta.carata@unisalento.it (E.C.); marco.muci@unisalento.it (M.M.); tiziano.digiulio@unisalento.it (T.D.G.); stefania.mariano@unisalento.it (S.M.); 2 Department of Mathematics and Physics, University of Salento, 73100 Lecce, Italy; simona.digiulio@unisalento.it

**Keywords:** amyotrophic lateral sclerosis, skeletal muscle, extracellular vesicles, biosensors, miRNA

## Abstract

Amyotrophic lateral sclerosis (ALS) represents a neurodegenerative disorder characterized by the progressive loss of both upper and lower motor neurons, resulting in muscular atrophy and eventual paralysis. While much research has concentrated on investigating the impact of major mutations associated with ALS on motor neurons and central nervous system (CNS) cells, recent studies have unveiled that ALS pathogenesis extends beyond CNS imbalances, encompassing dysregulation in other tissues such as skeletal muscle. Evidence from animal models and patients supports this broader perspective. Skeletal muscle, once considered solely as an effector organ, is now recognized as possessing significant secretory activity capable of influencing motor neuron survival. However, the precise cellular and molecular mechanisms underlying the detrimental effects observed in muscle and its associated structures in ALS remain poorly understood. Additionally, emerging data suggest that extracellular vesicles (EVs) may play a role in the establishment and function of the neuromuscular junction (NMJ) under both physiological and pathological conditions and in wasting and regeneration of skeletal muscles, particularly in neurodegenerative diseases like ALS. This review aims to explore the key findings about skeletal muscle involvement in ALS, shedding light on the potential underlying mechanisms and contributions of EVs and their possible application for the design of biosensors.

## 1. Introduction

Amyotrophic lateral sclerosis (ALS) stands out as the most prevalent motor neuron disease in adults. This condition is characterized by a relentless and rapid decline of upper motor neurons in the motor cortex and lower motor neurons in the brainstem and spinal cord. Clinical presentations of ALS encompass a spectrum of symptoms including dysarthria, dysphagia, spasticity, hyperreflexia, fasciculation, and sarcopenia [1]. Typically, respiratory failure ensues 2–5 years following symptom onset [2,3], representing the primary cause of mortality. Approximately half of ALS patients encounter cognitive and behavioral impairments, with a subset (5–25%) developing frontotemporal dementia (FTD), which manifests as profound alterations in the frontal and temporal lobes [4]. Despite its widespread occurrence, ALS holds a classification as a rare disease due to its dismal survival rates, rendering it one of the deadliest of illnesses [5]. The etiology of ALS remains that of an enigmatic disorder; approximately 10% of ALS cases, categorized as familial, exhibit a Mendelian inheritance pattern, characterized by autosomal dominant transmission. The remaining 90% of cases lack a discernible common cause and are thus classified as sporadic [6]. Over time, an array of mutations have been unearthed across a heterogeneous spectrum of genes, contributing to the pathogenesis of ALS. Among these, a pioneering discovery was the identification of mutations within the *SOD1* gene, encoding a ubiquitous Cu/Zn superoxide dismutase. Over 150 mutations within the *SOD1* gene have been catalogued, with these mutations implicated in around 20–25% of familial ALS (fALS) cases. Interestingly, both the wild-type *SOD1* gene and misfolded *SOD1* protein have been associated with certain sporadic ALS (sALS) cases [7]. Other prominent genes linked to ALS pathology include *C9orf72* (found in 39% of fALS cases and 7% of sALS cases), *TDP-43* (present in 4.2% of fALS cases and 0.8% of sALS cases), and *FUS* (detected in 2.8% of fALS cases and less than 1% of sALS cases) Presently, only two medications, namely riluzole and edaravone (the latter not universally approved), are available for potential therapeutic interventions. However, their efficacy is limited to slowing down disease progression without halting neuronal degeneration or muscle wasting.

Skeletal muscle wasting, also known as muscle atrophy, represents a significant physiological and pathological phenomenon characterized by a loss of muscle mass and function. This condition can arise from a variety of factors, including disease, ageing, malnutrition, chronic diseases such as cancer, heart failure, and chronic obstructive pulmonary disease (COPD), as well as neurological disorders like ALS and muscular dystrophy [8]. At the molecular level, muscle wasting involves dysregulation of multiple cellular processes, including protein degradation pathways such as the ubiquitin-proteasome system (UPS) and autophagy, as well as impaired protein synthesis. These dysfunctions lead to the accelerated degradation of contractile proteins and organelles, resulting in a net loss of muscle tissue. Additionally, pro-inflammatory cytokines and oxidative stress contribute to the progression of muscle wasting by promoting muscle protein breakdown and inhibiting muscle regeneration [9]. Understanding the intricate molecular mechanisms underlying skeletal muscle wasting is essential for the development of effective therapeutic strategies to combat this debilitating condition and improve the quality of life for affected individuals [8]. Generally, the decline in skeletal muscle mass stems from an imbalance between protein synthesis and proteolysis. Much attention has been directed towards the significance of proteolysis, highlighting the degradation of myofilaments as a primary driver of muscle wasting. Initially, intact myofibrillar proteins undergo degradation to release myofilaments from the myofibrillar structure, facilitating their subsequent breakdown. This cleavage of myofilaments is calpain-dependent, with the degradation of proteins anchoring myofilaments to the Z-disc enabling the separation of myofilaments [10]. The degradation of myofilaments via activated ubiquitin-proteasome and autophagy-lysosome pathways precipitates muscle wasting. Various inflammatory cytokines such as tumor necrosis factor α (TNF-α), interleukin-6 (IL-6), interleukin-1 α (IL-1α), and interferon-γ (IFN-γ), produced by either tumor or host cells, can serve as triggers for these pathways. These cytokines stimulate downstream signaling pathways including nuclear factor kappa-B (NF-κB) and p38 MAPK (p38 mitogen-activated protein kinase), subsequently activating forkhead family transcription factors that regulate crucial E3 ubiquitin ligase genes such as MuRF-1 (Muscle RING Finger containing protein 1, also known as TRIM63) and atrogin-1 (muscle atrophy F-box, also known as atrogin-1). These genes mediate the proteasomal degradation of muscle proteins and inhibit muscle protein synthesis. Additionally, autophagy genes can be activated through the signaling cascades initiated by these cytokines. Furthermore, members of the TGF-β (transforming growth factor- β) family, including activin A and myostatin, are implicated in promoting muscle wasting via the myostatin receptor ActRIIB.

Beyond the direct transduction of cytokine signals, emerging evidence suggests that factors associated with extracellular vesicles (EVs) can also induce a catabolic state in skeletal muscle [11,12]. In the muscle wasting process, EVs originating from parent cells transport a variety of functional biomolecules, which can have either a promoting or inhibiting effect on skeletal muscle wasting when absorbed by skeletal muscle cells [13].

EVs are small membranous structures released by cells into the extracellular environment. They are implicated in intercellular communication, transporting bioactive molecules such as proteins, lipids, and nucleic acids to recipient cells, thereby influencing various physiological and pathological processes [14]. EVs are commonly classified into three main categories based on their biogenesis and size: exosomes or small extracellular vesicles (SEVs), microvesicles or large extracellular vesicles (LEVs, also known as microparticles or ectosomes), and apoptotic bodies. Exosomes are small vesicles (30–150 nm) formed by the inward budding of endosomal membranes, leading to the formation of multivesicular bodies (MVBs) that release exosomes upon fusion with the plasma membrane. Microvesicles, on the other hand, are larger vesicles (100–1000 nm) that shed directly from the plasma membrane through outward budding. Finally, apoptotic bodies are larger vesicles (1–5 μm) released during programmed cell death (apoptosis), containing cellular remnants and organelles. Each type of EV exhibits distinct characteristics in terms of size, composition, and biogenesis, reflecting their diverse functions in intercellular communication [15].

Studies have shown that EVs are secreted by various cell types in the central nervous system (CNS), and recent research has shed light on the important roles EVs play in CNS function, including development, protection, repair, regulation of activity, and disease progression [16]. Neurally derived EVs are found in both blood and cerebrospinal fluid (CSF), with a higher concentration and specificity in the CSF [17]. While isolating EVs from CSF is challenging, detecting them in blood is a simpler yet effective method, though it may be complicated by the presence of diverse sources. Both CSF and blood EVs offer unique advantages for studying neurodegenerative disorders and their potential roles in pathology and diagnosis.

Neurodegenerative diseases, including ALS, often involve the misfolding, aggregation, and consequent accumulation of amyloid proteins in brain cells. Detecting these proteins in body fluids and tissues could aid in early disease diagnosis. Currently, there are no established therapies to effectively delay or prevent the progression of these diseases. Recent research has shown that EVs play a critical role in transmitting pathogenic protein aggregates between cells, which may contribute to the advancement of neurodegenerative diseases [18].

Here, we discuss the key findings about skeletal muscle disorder in ALS to shed light on potential underlying mechanisms and the contributions of EVs in communication between motor neurons and muscle cells; moreover, the possible application of EVs for the design of biosensors for diagnosis is reported.

## 2. Data Collection Methodology

Articles and reviews regarding amyotrophic lateral sclerosis and extracellular vesicles were identified from the Web of Science database and PubMed. Web of Science (WoS, Clarivate Analytics, Philadelphia, PA, USA), one of the most complete and accredited database platforms, contains more than 22,000 peer-reviewed journals. PubMed collects more than 37 million citations for biomedical literature from MEDLINE about life science journals and books. All the published papers were collected from a database, setting a period from January 2000 to February 2024. In this study, the search terms were shown as follows, using Boolean operators: TS = (amyotrophic lateral sclerosis) AND TS = (Extracellular Vesicles OR exosomes OR microvesicles OR microparticles OR ectosomes OR oncosomes OR apoptotic bodies) AND TS = (skeletal muscle) AND TS = (miRNA) AND TS = (biosensor). The publication inclusion criteria were as follows: (1) The manuscript focused on the theme of EVs based on amyotrophic lateral sclerosis and neuromuscular junction; (2) The document types were Article and Review. (3) The papers must have been written in English. The exclusion criteria were as follows: (1) The themes were not related to EVs based on amyotrophic lateral sclerosis; (2) Articles were briefings, news, meeting abstracts, etc.

## 3. The Role of Extracellular Vesicles in Neurodegenerative Disorders

Gradual loss of neurons due to the accumulation of abnormal proteins in both the central and peripheral nervous systems characterizes neurodegenerative diseases, such as Alzheimer’s disease (AD), Parkinson’s disease (PD), Huntington’s disease (HD), and amyotrophic lateral sclerosis, whose causes are complex and not yet completely known [19]. Nonetheless, both the transfer of harmful molecules between different cell populations and the disease-related environment have been identified as significant factors in the development of these diseases. Cells can communicate with each other through multiple pathways, but EVs are considered to be one of the most effective methods of intercellular communication [20].

So far, numerous studies have documented the involvement of EVs in the development and advancement of AD, PD, ALS, and HD as well as the role of miRNAs-delivered EVs as biomarkers [21] Below is how molecules carried by EVs contribute to the pathogenesis of AD, PD and HD. Conversely, the role of extracellular vesicles in ALS is reported in Section 4.

AD is characterized by a gradual decline in cognitive function and the presence of plaques formed by aggregates of Aβ peptides produced upon the cleavage of amyloid precursor protein (APP), and tangles containing hyperphosphorylated Tau protein that accumulate in axons and dendrites [22]. Additionally, other hypotheses such as neuroinflammation [23], gut–brain axis disorder [24], and metabolic dysfunction [25] have been suggested. It is worth noting that studies have indicated changes in the secretion and roles of EVs throughout the advancement of Alzheimer’s disease, as plasma EVs may play an important role in the communication between the brain and the periphery, potentially accelerating the progression of AD. In fact, halting the release of EVs has been shown to greatly reduce the symptoms of AD, underscoring the significant impact of EVs on the development of the disease [26].A summary of pathological functions of EVs in AD is reported in Table 1.

Clinical signs of PD include the progression of rigidity, bradykinesia, and tremor due to the loss of dopaminergic neurons in the substantia nigra, resulting in decreased dopamine levels in the striatum and the presence of Lewy bodies in remaining neurons [39]. The accumulation of α-synuclein (α-syn) is implicated in neurodegeneration and contributes to PD symptoms. EVs have emerged as potential contributors to PD for their ability to transport α-syn [40]. Research has also shown that cerebrospinal fluid EVs from PD patients can induce α-syn aggregation in target cells, potentially contributing to disease progression. In vitro and in vivo experiments suggesting the link between EVs and PD in the transmission of molecules impacting the pathophysiology of PD are listed in Table 2.

HD is characterized by progressive chorea, oculomotor abnormalities, verbal ataxia, dysphagia, dementia, depression, anxiety, and apathy due to the loss of long-projection neurons in the cortex and striatum. HD is inherited in an autosomal dominant manner due to CAG repeats (≥36) in the Huntington’s disease chorea gene (*IT15*) on chromosome 4, resulting in an abnormal number of N-terminal glutamine repeats (polyQ) in mutated huntingtin protein (mHTT) [48]. EVs cause a shuttle of mHTT in HD, leading to mitochondrial dysfunction and ultimately cell death in recipient cells [49]. In Table 3, we report a summary of EV contents and their involvement in HD.

## 4. The Role of Extracellular Vesicles in Amyotrophic Lateral Sclerosis

EVs have emerged as key players in the pathogenesis of amyotrophic lateral sclerosis (ALS), a progressive neurodegenerative disorder characterized by the selective degeneration of motor neurons in the brain and spinal cord. Mounting evidence suggests that EVs, including exosomes and microvesicles, contribute to the propagation of pathological proteins, neuroinflammatory signals, microRNAs and lipids, thereby exacerbating disease progression in ALS.

EVs can transport misfolded proteins, such as mutant superoxide dismutase 1 (SOD1), TAR DNA-binding protein 43 (TDP-43) or FUS, implicated in ALS pathogenesis, facilitating their intercellular spread and seeding of protein aggregation in recipient cells [52,53].

The transportation of TDP-43 via exosomes is pathologically significant, as ALS patient brains and CSF showed significantly elevated levels of exosomal full-length (FL) and C-terminal fragment (CTF) TDP-43 compared to controls [54,55]. Wildtype, mutant, and CTF TDP-43 can be found in exosomes, and while neuron-like cells and primary neurons secrete TDP-43 via exosomes, astrocytes and microglia do not. Despite conflicting evidence, this suggests that once internalized, TDP-43 delivered by EVs can induce cytoplasmic redistribution and aggregation of native TDP-43, leading to stress, redistribution, and aggregation, perpetuating the progression of the disease.

Numerous studies have demonstrated the intercellular transmission of misfolded SOD1 through extracellular vesicles, particularly exosomes. Unlike the natively folded SOD1, which is located within the exosomal lumen, misfolded SOD1 is found on the surface of vesicles, as shown by Grad et al. [56] and Silverman et al. [53]. The most compelling evidence supporting the role of EVs in the spread of SOD1 comes from the discovery of misfolded and aggregated SOD1 in EVs obtained from astrocytes and neurons of the brains and spinal cords of SOD1-ALS patients and SOD1-G93A mice [53]. In contrast to Silverman’s findings, Massenzio et al. [57] and Vaz et al. [58] showed that also microglia release SOD1 via exosomes. Moreover, in co-culture experiments, microglia with mutant SOD1-G93A or SOD1-A4V induced toxicity in primary cerebellar granule neurons, while astrocytes did not show the same effect [57]. However, there are still several unanswered questions about the transmission of SOD1 aggregates through exosomes. These include the specific mechanisms by which SOD1 is included in exosomes and how recipient cells uptake exosomal SOD1. The significance of extracellular vesicles in spreading SOD1 pathology in human ALS remains unclear.

Recently, Carata and coworkers [59] studied the molecular profile of EVs released by mSOD1 NSC-34 motor neuron-like cells. EVs carry to Raw 264.7 macrophages the pro-inflammatory cytokines IL-1β, IL-6 and TNFα, the chemoattractant factor MIF-Macrophage migration inhibitory factor, and the aggregates of the mutated TDP43 protein and mSOD1, able to cause M2b activation sustaining a both protective and pathogenic environment.

Additionally, secreted EVs contain other ALS-related mutated proteins, albeit at a lower concentration compared to SOD1 and TDP-43. Mutations in FUS can result in varied mislocalization of the protein in the cytoplasm, potentially contributing to the formation of stress granules. Kamelgarn first demonstrated the presence of FUS in EVs, supporting the idea of FUS spreading between pathological cells and also suggesting a potential diagnostic tool for ALS [60].

Finally, the spread of dipeptide repeat (DPR) through EVs in ALS motor neurons with C9orf72 has been observed [61].

EVs derived from ALS patient-derived cells or biofluids exhibit altered cargo composition, including aberrant levels of microRNAs, which may influence neuronal viability and contribute to disease pathogenesis. The miRNA profiles in EVs in the plasma of ALS patients show significant alterations. For example, in ALS patients, 13 miRNAs were up-regulated, and 17 miRNAs were down-regulated compared to controls, affecting neuroplasticity and neural viability [62]. Dysregulated miRNAs found in EVs from ALS patients, such as miR-494-3p and miR-124, impact axonal development, neuronal survival, and inflammation [63]. Furthermore, astrocytes derived from ALS patients with C9orf72 mutations release EVs that do not contain miR-494-3p, a molecule that normally suppresses the expression of the semaphorin 3 A (SEMA3A) gene, which is important for maintaining axons. The absence of miR-494-3p in EVs allows SEMA3A to contribute to the degeneration of motor neurons in ALS [64]. Finally, levels of miR-155, and miR-146a are notably elevated in EVs originating from mSOD1 microglia. miR-155 and miR-146a are recognized as pro-inflammatory microRNAs that modulate microglial activation [58].

Additionally, EVs released by glial cells, including astrocytes and microglia, can modulate neuroinflammatory responses and neurotoxicity in ALS through the delivery of pro-inflammatory cytokines, reactive oxygen species, and other cytotoxic factors to neighboring neurons. Chen et al. demonstrated a notable rise in IL-6 levels in EVs obtained from the plasma of sporadic ALS patients, highlighting the crucial role of EV-mediated dissemination of pro-inflammatory substances in the onset and progression of neuroinflammation, a pivotal aspect of ALS pathology [65]. Vaz demonstrated the increase in the levels of HMGB1 in mSOD1 microglia-derived EVs that can elicit neuroinflammation by disrupting mitophagy flux [58]. Neural stem cell-derived EVs overexpressing the insulin-like growth factor 1 receptor have been shown to alleviate neurologic impairments in ALS mouse models by enhancing neuronal survival and reducing neuroinflammation [66]. Table 4 summarizes EV miRNA and proteins involved in ALS pathogenesis.

The ability to transfer miRNAs makes EVs also potential biomarkers for ALS. miR-124-3p was demonstrated by Yelick as involved in the staging of disease and suggested as a valuable prognostic biomarker for ALS [70]. Rizzuti and colleagues [71] identified miR-34a, miR-335, and miR-625-3p in cerebrospinal fluid-derived EVs as potential biomarkers for ALS. Meanwhile, Freischmidt et al. [72] analyzed miRNA levels in EVs from the CSF and serum of 22 sporadic ALS patients and 24 healthy individuals. They found that patients with ALS had significantly lower levels of miR-132-5p, miR-132-3p, and miR-143-3p in EVs, while miR-143-5p and miR-574-5p levels were significantly higher, suggesting their potential as biomarkers for ALS detection. Xu et al. [73] discovered a significant decrease in miR-27a-3p levels in serum EVs of ALS patients, suggesting its potential as a diagnostic biomarker. Lo et al. [74] showed that various miRNAs, e.g., miR-342-3p, miR-1254, miR-587, miR-298, miR-4443 and miR-450a-2-3p, in serum and brain tissue-derived EVs can indicate the state of CNS disease in ALS, offering a possibility for diagnosis. Gomes et al. [75] identified inflammatory-related miRNAs miR-155, miR-21, and miR-146a as depleted in EVs from both spinal and cortical astrocytes in ALS mice, indicating their potential use as ALS biomarkers.

In addition to the cargo of nucleic acids carried by EVs, a study conducted by Morasso pioneered the investigation into distinct lipid profiles within EVs derived from sporadic sALS patients. Through the utilization of Raman spectroscopy, they unveiled distinct biochemical fingerprints of LEVs present in the plasma of sporadic ALS patients compared to healthy controls. Notably, LEVs from ALS patients exhibited a heightened abundance of lipids while showing a depletion in phenylalanine content, potentially reflecting alterations in the protein composition of LEVs. Conversely, SEVs displayed less pronounced differences in lipid composition compared to LEVs. Importantly, the findings from this study highlight the potential utility of LEVs as promising diagnostic biomarkers for ALS, shedding light on the involvement of lipid metabolism and phenylalanine pathways in LEVs derived from sALS patients, thus offering insights into ALS pathogenesis [76].

Furthermore, EVs may participate in non-cell-autonomous mechanisms of motor neuron degeneration by promoting astrocyte activation, impairing the blood–spinal cord barrier integrity, and facilitating immune cell infiltration into the central nervous system. Overall, elucidating the complex roles of EVs in ALS pathophysiology holds promise for identifying novel diagnostic biomarkers and therapeutic targets aimed at mitigating neuroinflammation and neuronal dysfunction in this devastating neurodegenerative disease [77].

## 5. The Neuromuscular Junction and Possible Alteration

The neuromuscular junction (NMJ) serves as a critical interface between the nervous system and the muscular apparatus, orchestrating the intricate network of signal transmission that underlies every voluntary muscle movement. A spectrum of disorders affecting this junction have emerged as a focal point of scientific inquiry due to their profound impact on motor function and overall physiological homeostasis. One of the crucial events in the development of ALS at the muscle level may be the damage of the NMJ [78,79,80]. Functional and morphological changes have been identified across various components of the NMJ in ALS patients and different in vivo ALS models. These alterations, as the disease progresses, lead to destabilization and disassembly of the NMJ. Recent research has demonstrated that NMJ denervation in ALS is a multifaceted and dynamic process involving continuous denervation and re-innervation at the level of individual motor units, rather than an immediate consequence of motor neuron degeneration. Studies in SOD1G37R mice by Martineau revealed asynchronous dismantling of motor units, with gradual local initiation followed by abrupt global axonal degeneration [81]. Additionally, the denervation process is initially countered by a re-innervation process that persists until it becomes unsustainable to offset the initial degeneration. Tallon observed in SOD1 mice that axonal degeneration follows a length-dependent pattern, with longer axons innervating caudal regions being more susceptible. In ALS model animals, changes in the synaptic properties of specific motor units precede morphological alterations of the NMJ and motor neuron degeneration [79].

**Figure 1:**
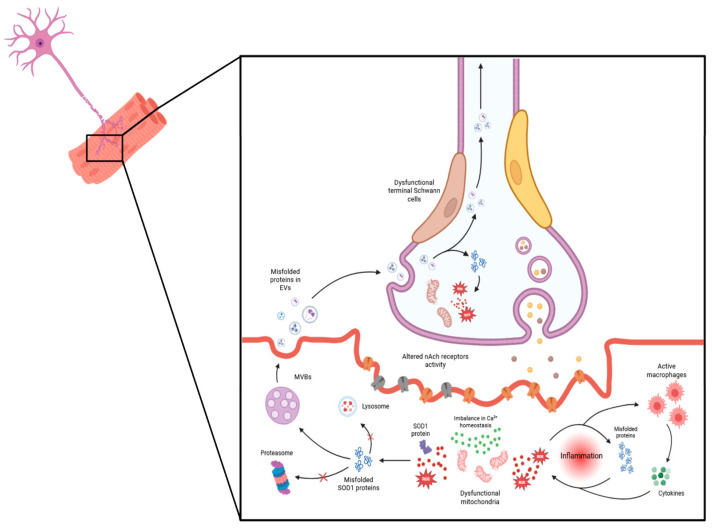
Cellular mechanisms underlying neuromuscular junction (NMJ) dismantling in amyotrophic lateral sclerosis (ALS). Key events include mitochondrial dysfunction, accumulation of reactive oxygen species (ROS), disrupted calcium (Ca^2+^) homeostasis, and oxidative stress, all of which contribute to an inflammatory environment. Meanwhile, disruptions in protein homeostasis, indicated by changes in protein synthesis and degradation, contribute to the development of protein aggregates, such as superoxide dismutase 1 (SOD1). These aggregates exacerbate tissue damage over time, leading to skeletal muscle atrophy. Additionally, extracellular vesicles released from the muscle propagate toxic factors to other NMJ components, thereby destabilizing the NMJ and leading to a progressive decline in motor function. MicroRNAs (miRNAs), loaded in EVs, also play a role in impairing neuromuscular junctions (NMJs) by effectively regulating muscle homeostasis, plasticity, and myogenesis under both normal and pathological conditions. They are crucial for the formation and maturation of neuromuscular synapses, and their dysregulation has been implicated in the degeneration observed in ALS. For instance, miR-206 shows increased expression in ALS model patients and animals, correlating with disease progression in the symptomatic stage, while miR-133b and miR-1 exhibit dysregulated levels in skeletal muscle, spinal cord, and muscle biopsies from ALS patients [82]. Conversely, decreased expression of miR126-5p in the skeletal muscle of ALS mouse models contributes to ALS pathology by promoting axonal degeneration and NMJ destruction, possibly through elevated expression and secretion of semaphorin 3A. Notably, the overexpression of miR126-5p in vitro in myocytes carrying the SOD1G93A mutation and in SOD1G93A mice has been shown to transiently alleviate the effects of axonal degeneration and NMJ destruction and inhibit the neurodegenerative process [83].

Figure 1 delineates the potential cellular mechanisms implicated in the dismantling of the neuromuscular junction (NMJ) in ALS, illustrating several interrelated processes that contribute to ALS pathogenesis.

## 6. Muscle Cell Differentiation and Regeneration Mediated by Extracellular Vesicles

Skeletal muscle constitutes about 35% of total body weight, and this tissue is continually exposed to contraction with consequent ageing damage of sarcolemma, which compromises efficiency, particularly during physical exercise [84]. The damaged muscle cells recruit the satellite cells to promote the regeneration process. This mechanism needs numerous events such as inflammation, neovascularization, innervation, muscle proliferation, and differentiation mediated by inflammatory cytokines, miRNA, proteins, and EVs released by myoblast cells [85]. In the context of muscle biology, EVs have emerged as important mediators of myoblast differentiation. During the process of muscle differentiation, myoblasts undergo molecular and structural changes to become multinucleated myotubes, which eventually mature into functional muscle fibers. Several studies, carried out using C2C12 cells, have demonstrated that the secretion of EVs increases concomitantly with the progression of myoblast differentiation. These EVs are enriched with various growth factors, including basic fibroblast growth factor (bFGF), insulin-like growth factor-1 (IGF-1), transforming growth factor-beta1 (TGF-B1), vascular endothelial growth factor (VEGF), and others. Importantly, these growth factors serve as potent regulators of muscle development, function, and repair. The paracrine activity of EV-derived growth factors is particularly significant in orchestrating muscle satellite cell chemotaxis and lineage commitment, crucial events in muscle regeneration and repair. Furthermore, studies have shown that EVs derived from myotubes possess the capacity to promote the differentiation of myoblasts by modulating the expression of key regulatory proteins involved in myogenesis. These findings underscore the intricate role of EVs in coordinating the molecular events underlying muscle differentiation and highlight their potential therapeutic implications in muscle regeneration and repair strategies [86]. During muscle regeneration, miRNAs transported by EVs act as key mediators in modulating the expression of genes involved in myoblast proliferation, differentiation, and fusion (Table 5). For instance, specific miRNAs, such as miR-1, miR133a,b, and miR206, packaged within EVs have been shown to target and regulate the expression of key transcription factors and signaling molecules essential for myogenic differentiation, such as myogenic regulatory factors (e.g., MyoD, Myogenin) and Wnt/β-catenin pathway components. Moreover, EV-delivered miRNAs have been implicated in regulating the inflammatory response, angiogenesis, and extracellular matrix remodeling, processes crucial for efficient muscle repair. Importantly, the selective packaging of miRNAs into EVs suggests a sophisticated mechanism for fine-tuning gene expression in recipient cells, thereby exerting precise control over the molecular events underlying muscle regeneration. Understanding the repertoire and functional significance of miRNAs carried by EVs in the context of muscle regeneration holds promise for the development of novel therapeutic strategies aimed at enhancing muscle repair and functional recovery following injury or degenerative disorders [87].

**Table 5:** Summary of miRNAs carried by EVs and involved in muscle regeneration.

miRNA in EVs	Biological Effect	Target Genes	Ref.
miR-501	Inflammation decrease	YY1	[88]
miR-148a	Myogenic differentiation	ROCK-1	[89]
miR-224	Myogenic differentiation and proliferation	WNT-9a	[90]
miR-1	Skeletal muscle proliferation, differentiation and regeneration	BDNF, CCND1, CCND2, FZD7, G6PD, GJA1, HACD3, HDAC4, HSPA1, IGF1, IGF1R, MAP4K3, MEOX2, MET, MMD, NFAT5, NOTCH3, PAX3, PAX7, POLA1, RARB, SARS, SMARCB1, SMARCD2, UTRN, VEGFA, YY1	[91,92]
miR-133a	Myoblast proliferation, differentiation and fusion, regeneration	CALM1, DNM2, FGFR1, FOXL2, IGF1R, MAML1, PFN2, PP2AC, PRDM16, PTBP2, RUNX2, SMARCD1, SP1, SRF, TRPS1, UCP2	[91,92]
miR-133b	Myoblast differentiation and fusion	FAIM, FGFR1, MAML1, PP2AC, PRDM16, PTBP2, SP1	[91,92]
miR-206	Myoblast differentiation, regeneration, regeneration of neuromuscular synapses	BDNF, CCND1, CCND2, CLCN3, FSTL1, FZD7, G6PD, GJA1, HACD3, HDAC4, HMGB3, IGF1, IGFBP5, MAP4K3, MEOX2, MET, MMD, NFAT5, NGFR, NOTCH3, PAX3, PAX7, POLA1, RARB, SH3BGLR3, SMARCB1, SMARCD2, SNAI2, TIMP3, UTRN, VEGFA	[91,92,93]
miR-208b	Muscle fiber shift, promotion of muscle growth	CBX1, MED13, MSTN, PURB, SOX6, SP3	[91]
miR-486	Myoblast differentiation and fusion	DOCK3, FOXO1, PAX7, PDGFRB, PTEN, SRSF1, SRSF3	[91]
miR-499	Muscle fiber shift, muscle growth promotion	CBX1, MAPK6, MED13, MSTN, PURB, SOX6, SP3	[91]
miR-126, miR-23a, miR494	Myogenesis and angiogenesis	MYO G, MYO D	[94,95]
miR29c	Muscle regeneration	TGFβ	[96]

## 7. A Possible Application of EVs in ALS: Biosensor-Based Detection of Exosomal miRNA

**Figure 2:**
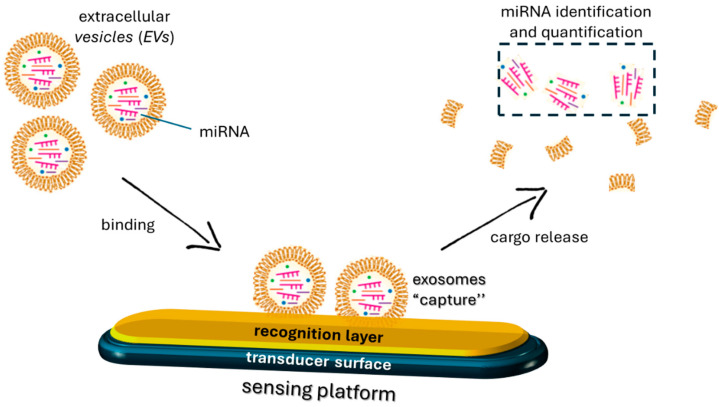
Schematic of a mechanism of exosome recognition by a sensing platform.

Recent studies have demonstrated that exosomal miRNAs can significantly impact myogenic differentiation by modulating cellular activities such as myoblast proliferation, differentiation, fusion, and muscle fiber maturation [97]. For example, miR-1 and miR-206 have been found to promote myogenic differentiation by targeting HDAC4, a negative regulator of muscle differentiation [89], while miR-133 inhibits muscle differentiation by targeting serum response factor (SRF), a key regulator of muscle-specific gene expression [98]. These molecules contribute to the regulatory network controlling muscle development, and their dysregulation can have implications for muscle-related diseases, such as muscular dystrophy or muscle wasting conditions. Moreover, the specific functions of miRNAs can vary depending on the context of muscle development and the stage of myogenesis [99]. These findings suggest the potential of these molecules in developing effective therapies for muscle disorders and provide insight into the complex mechanism of intercellular communication [71]. The encapsulation of miRNA into exosomes serves as a protective mechanism for these molecules, preventing their degradation by extracellular RNases. Additionally, it facilitates the targeted delivery to specific cells, allowing for intercellular communication and modulation of gene expression [100]. While the detailed mechanisms of sorting are still an important area of research, it has been proposed that the presence of specific motifs or sequences, often referred to as “EXO motifs” or “exosomal targeting sequences”, plays a role in guiding miRNAs into exosomes [101]. Recent research has shown that mature miRNAs contain specific motifs at their 3′-end, including uridine-rich sequences. These EXO motifs are thought to interact with two RNA-binding proteins, heterogeneous nuclear ribonucleoprotein A2B1 (hnRNPA2B1) and Y-Box protein 1 (YBX1), which act as chaperones and regulate the recognition and transport of miRNAs in exosomes. Other key factors such as the endosomal sorting complexes required for transport (ESCRT) machinery, the lipid composition of the exosomal membrane, and interactions with tetraspanins may also contribute to this process. Increasing evidence suggests that the packaging of miRNAs in exosomes is related to the presence of specific EXOmotif sequences following muscle-damaging stimuli [102]. For instance, activation of muscle satellite cells (SCs), in response to muscle injury, leads to exosomal release of the CCCG-EXOmotif containing miR-206, involved in SC differentiation and muscle regeneration [103]. Similarly, muscle damage associated with Duchenne muscular dystrophy (DMD) stimulates the release of miR-1 containing the GGAC-EXOmotif, miR-206 containing CCCG, and miR-133a containing the CCCU sequence, which has a pro-survival effect [104,105]. Their tissue-specific expression patterns make EXOmotifs and, more in general, miRNAs promising biomarkers for both diagnostic and prognostic interventions [106]. Monitoring these molecules can occur through their direct detection in biological fluids or by capturing the vesicles containing them [107,108,109,110,111]. The latter is particularly interesting, as exosomes are regarded as biomarkers with enormous potential for diagnosing, tracking, and treating various diseases. Exosomes are present in almost all body fluids, with their cargoes changing under physiological or pathological conditions, making them ideal as cell signaling mediators and sources of diverse biomarkers. Conventional exosome isolation methods are often expensive and inefficient, yielding vesicle-enriched solutions but containing unwanted components [109,112]. Developing sensors/biosensors could overcome these limitations, facilitating the widespread use of liquid biopsy-based analysis in clinical practice, although this has only been sporadically explored. In one example [109], sensing platforms were developed by functionalizing silicon oxide substrates with different receptors to capture exosomes. Functional assays were conducted to test these sensors using both exosome-mimicking vesicles and exosomes isolated from cell supernatants. The various surfaces exhibited promising properties, with the negatively charged surface capable of capturing over 4 × 10^8^ exosomes per square centimeter. The captured exosomes were recovered, and their biomarker cargoes were analyzed (Figure 2).

Direct detection of miRNAs released into biological fluids has been more extensively explored [107,108,111]. However, their full potential as biomarkers seems incompletely realized, perhaps due to inherent features such as short sequence length, low abundance, high sequence similarity, and wide range of expression levels, making their detection challenging [113]. Currently, conventional detection methods, including quantitative RT-PCR, next-generation sequencing (NGS), DNA microarray-based analysis, and northern blot rely on time-consuming and costly procedures that require specialized laboratory equipment, limiting their application in routine clinical practice. These limitations have driven the development of reliable biosensing platforms, which provide high sensitivity and specificity for targets, with significant advantages in terms of cost-effectiveness and rapid response, making them suitable for high-throughput applications [114]. The identification of myogenic-related exosomal miRNAs through the integration of sensing technologies targeting exposed regions is attracting considerable attention, offering valuable insights into the molecular processes involved in muscle-related disorders, thereby facilitating the development of targeted interventions [115]. To capture these molecules, biosensors typically employ specific oligonucleotide-labeled probes that are designed to hybridize with the target miRNA and then used as receptors to mediate the interaction with the target analyte. The recognition event produces a modification of a physical quantity that a transducer converts into an easily measurable signal (e.g., optical or electrochemical). The choice of probe sequences and labeling strategies can affect the sensitivity, selectivity, and stability of the biosensor; thus, these parameters need to be carefully optimized for each application [114]. In a recent study conducted by Kang et al., fluorescent-based molecular beacons (MBs) were developed for the detection of mir-26a and mir-206 during the myogenesis of C2C12 cells, both in vitro and in vivo [111]. MBs are synthetic oligonucleotide probes with a stem-loop (hairpin) structure that undergoes a conformational transition in the presence of the target sequence. The probe sequence, specific to the target nucleic acid, is coupled with a fluorophore moiety and a quencher. When the molecular beacon encounters its target sequence, it undergoes a conformational change, opening the hairpin structure and producing a spatial separation between fluorophore and quencher, leading to an increase in fluorescence signal, used as an analytical parameter to monitor the target [116]. In another example [107], an electrochemical biosensor was developed for the specific recognition of miR-206 from plasma samples. The process involved the anchoring of the molecular recognition system on the electrode surface able to recognize the target miRNA. The binding of the target on the electrode surface by the receptor produces a change in the current signal, used to detect the analyte. Recently [108], surface plasmon resonance (SPR) biosensors for miRNA detection were also developed. In this case, changes in the refractive index on the sensor surface, resulting from the binding or detachment of target molecules, were used for analyte monitoring. The sensors were employed to detect miR-122 from total RNA samples extracted from humans. Specifically, the chip surface was functionalized with covalently attached thiolated DNA probes, with a short complementary sequence for the recognition of the miRNA. Moreover, streptavidin-linked oligonucleotides were added to the sensor surface to specifically bind another section of the target sequence, allowing for signal amplification. Sensors offer distinct advantages over other detection techniques for miRNA molecules. They provide rapid and sensitive detection of miRNA levels with high specificity. Additionally, sensors are versatile and can be tailored to detect specific miRNA sequences, facilitating personalized diagnostics. Furthermore, sensor-based detection is non-invasive, requiring small sample volumes, which is advantageous for clinical applications [117,118].

## 8. Conclusions

This review highlights how extracellular vesicles can be exploited in ALS with particular attention to the impact on muscle regeneration. Muscle wasting in skeletal muscles characterizes ALS and significantly impacts quality of life, leading to weakness, paralysis of the muscles, and eventually death. Numerous studies have focused on the molecular mechanisms behind muscle wasting, with particular attention to the role of EVs in this process. Multiple studies have shown a strong connection between EVs and neurodegenerative diseases, including ALS. This includes EVs transporting harmful molecules, influencing immune cell responses, regulating neuronal function, and maintaining blood–brain barrier integrity. These findings have tremendous potential in the field of translational medicine. Furthermore, a growing number of clinical trials have explored the practical applications of EVs in neurodegenerative diseases. For instance, a clinical investigation was carried out to identify biomarkers for PD susceptibility and/or progression using exosome-proteomes derived from PD patients (ClinicalTrials.gov Identifier: NCT01860118). Another study assessed the safety and effectiveness of exosomes derived from allogeneic adipose mesenchymal stem cells in individuals with AD (ClinicalTrials.gov Identifier: NCT04388982). To date, there are no clinical trials on ALS and extracellular vesicles in the database ClinicalTrials.gov. As indicated by the investigations discussed in this review, with the advancement of research in this field, EVs have shown promise in applications for the diagnosis and/or treatment of neurodegenerative diseases.

The content of vesicles can influence the inflammatory, regenerative, and remodeling processes, indicating that EVs could potentially be utilized as a therapeutic approach to enhance skeletal muscle regeneration. In particular, miRNAs enclosed in EVs have important effects on muscle regeneration. EV-encapsulated miRNAs and proteins have been identified as key players in promoting or preventing muscle loss in several diseases, and the findings can be exploited also in the management of ALS. Knowledge of EV function in muscle wasting could lead to the development of diagnostic biomarkers and innovative treatment strategies. Utilizing EVs as drug carriers and delivery systems shows promise in attenuating muscle wasting, but more research is required to optimize their effectiveness. Targeting EV-associated pro-wasting cargoes or EV biogenesis itself may offer a promising approach to combat EV-induced muscle loss.

**Figure 3:**
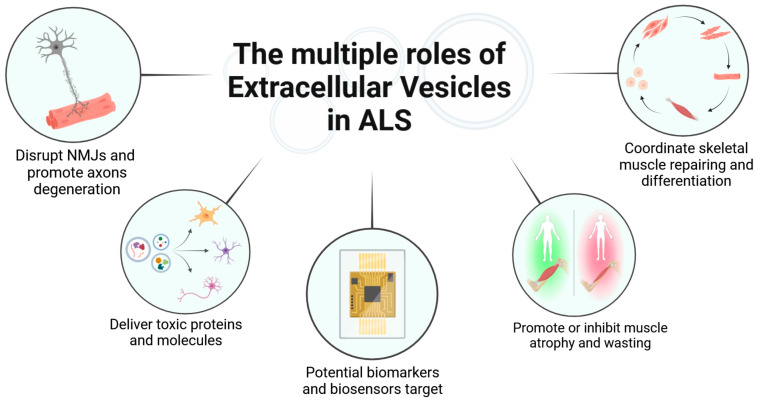
Overview of the roles of extracellular vesicles in ALS (Created in BioRender).

Figure 3 is a graphical summary of the roles of EVs in ALS as reported in the text.

In conclusion, the involvement of EVs in ALS and muscle differentiation, regeneration, and atrophy underscores the intricate interplay between cellular communication and pathological processes. These vesicles serve as vital mediators, orchestrating signaling cascades that influence disease progression and muscle homeostasis. Understanding the roles of EVs in these contexts holds promise for unraveling novel therapeutic or diagnostic avenues and enhancing our comprehension of the complex mechanisms underlying ALS and muscular disorders.

## Figures and Tables

**Table 1:** The biological effects of EV cargoes in Alzheimer’s disease.

Cargo	Biological Effect on Recipient Cells	Ref.
p-tau 181, p-tau 396, Aβ_1-42_, cathepsin D	Aβ deposition and NFT formation promotion	[27,28,29]
Human Tau	Tau protein spreading	[30]
APP	Promotion of Aβ production	[31]
BACE1, complement proteins	Aβ cleavage and neuronal damage	[32,33]
TREM2	Aβ accumulation in microglia	[34]
miR-124, miR-155, miR-146a, miR-21, miR-125b	Microglia activation Pro-inflammatory cytokine release	[35]
miR-185	Up-regulation of APP expression	[31]
miR-28-5p, miR-381-3p, miR-651-5p, miR-188-5p	Neuroinflammation and senescence increase	[36]
miR-16-5p, miR-451a, miR-605-5p	Neuron projections, synaptic signaling, apoptosis, and immune system modulation	[37]
miR126-3p, miR142-3p, miR-223-3p	Neural damage, specific miRNA downregulation, and inflammatory cytokine upregulation	[38]

NFT, Neurofibrillary tangles; APP, amyloid precursor protein; BACE1, β-site APP cleaving enzyme 1 or β-secretase; TREM2, Triggering receptor expressed on myeloid cells 2.

**Table 2:** The biological effects of EV cargoes in Parkinson’s disease.

Cargo	Biological Effect on Recipient Cells	Ref.
α-syn	Neurotoxicity induction α-syn-rich Lewy bodies formation Spreading α-syn from microglia to neurons	[41,42]
MHC II, TNFα	Apoptosis induction Neuroinflammation induction Dopaminergic neurodegeneration	[43]
Rab8b, Rab31	Hearing loss	[44]
Gelsolin, PARK7, 20S proteasome complex	PD onset and progression	[45]
miR-200a-3p	Inhibition of cell death	[46]
miR-19a-3p, miR-155	Inflammation induction	[47]

MHC II, major histocompatibility complex class II; TNFα, tumor necrosis factor α; PARK7, parkinson protein 7.

**Table 3:** The biological effects of EV cargoes in Huntington’s disease.

Cargo	Biological Effect on Recipient Cells	Ref.
CSPα	Synaptic proteostasis	[50]
mHTT	Neurodegeneration	[49]
CRYAB	EVs containing mHTT release suppression	[51]

CSPα, cysteine string protein; mHTT, mutant huntingtin protein; CRYAB, Alpha-crystallin B.

**Table 4:** The biological effects of EV cargoes in amyotrophic lateral sclerosis.

Cargo	Biological Effect on Recipient Cells	Ref.
mSOD1	Motor neuron cell death Mitochondrial damage General neurotoxicity	[57,67,68]
DPRs, TDP-43	Astrocyte toxicity, neurodegeneration	[69]
IL-6	Inflammation	[65]
HMGB1	Microglia activation Mitophagy alteration	[58]
miR-24-3p, miR-1268a, miR-3911, miR-4646-5p, miR-939-5p, miR-3619-3p, miR-4298, miR-4700-5p, miR-4736, miR-4739, miR-1207-5p, miR-4505, miR-149-3p, miR-2861, miR-4508, miR-4507, miR-3176, miR-3911, miR-150-3p	Neuroplasticity alteration, Motor neuron damage	[62]
miR-155, miR-146a	Microglia activation	[58]
miR-124	Reduction of cell phagocytic ability of microglia Mixed M1 and M2 subpopulations of microglia Microglia senescence	[68]

mSOD1, mutated superoxide dismutase 1; DPRs, dipeptide repeats; TDP-43, TAR DNA-binding protein 43; IL-6, interleukin 6; HMGB1, High Mobility Group Box 1.

## Data Availability

Not applicable.
